# Biomolecular Monitoring Tool Based on Lab-on-Chip for Virus Detection

**DOI:** 10.3390/bios13050544

**Published:** 2023-05-12

**Authors:** Francesca Costantini, Nicola Lovecchio, Manasa Nandimandalam, Ariana Manglli, Francesco Faggioli, Mara Biasin, Cesare Manetti, Pio Federico Roversi, Augusto Nascetti, Giampiero de Cesare, Domenico Caputo

**Affiliations:** 1 CREA Research Centre for Plant Protection and Certification, 00156 Rome, Italy; 2 Department of Environmental Biology, Sapienza University of Rome, 00185 Rome, Italy; nicola.lovecchio@uniroma1.it (N.L.);; 3 Department of Information Engineering, Electronics and Telecommunications, Sapienza University of Rome, 00184 Rome, Italy; 4 Department of Biomedical and Clinical Sciences, University of Milan, Via G.B. Grassi, 20122 Milan, Italy; 5 School of Aerospace Engineering, Sapienza University of Rome, 00138 Rome, Italy

**Keywords:** Lab-on-Chip, real-time RT-PCR, System-on-Glass, virus detection

## Abstract

Lab-on-Chip (LoC) devices for performing real-time PCR are advantageous compared to standard equipment since these systems allow to conduct in-field quick analysis. The development of LoCs, where the components for performing the nucleic acid amplification are all integrated, can be an issue. In this work, we present a LoC-PCR device where thermalization, temperature control and detection elements are all integrated on a single glass substrate named System-on-Glass (SoG) obtained using metal thin-film deposition. By using a microwell plate optically coupled with the SoG, real-time reverse transcriptase PCR of RNA extracted from both a plant and human virus has been carried out in the developed LoC-PCR device. The limit of detection and time of analysis for the detection of the two viruses by using the LoC-PCR were compared with those achieved by standard equipment. The results showed that the two systems can detect the same concentration of RNA; however, the LoC-PCR performs the analysis in half of the time compared to the standard thermocycler, with the advantage of the portability, leading to a point-of-care device for several diagnostic applications.

## 1. Introduction

Quantitative real-time polymerase chain reaction (qPCR) is a molecular reaction used to specifically detect and quantify a segment of DNA through its amplification. qPCR is the gold standard technique for the detection of nucleic acid in many fields including diagnostics and medicine [1], agricultural science [2] and food science [3]. One of the advantages of qPCR is based on the elimination of the post-amplification steps such as electrophoresis for revealing the PCR product. This is feasible because in the reaction mixture is present a fluorophore whose fluorescent intensity is proportional to the amount of amplified DNA sequence and the PCR master mix containing the enzyme polymerase, the specific oligonucleotide, the primers and probe, the free nucleic acids and the salts, necessary for the duplication of the DNA segments. The PCR reaction occurs through the repetition of thermal cycling, which permits the polymerase enzyme to bind the sequence and to catalyze the formation of the new copies of the specific oligonucleotide sequence. The PCR reaction in the real-time mode is also applied for the detection of the RNA. In this case, the reverse transcriptase enzyme (RT) is added to the PCR mixture. RT transcripts the RNA to complementary c-DNA, which is then amplified via the real-time qPCR reaction. Real-time RT-qPCR is widely used for the detection of viral RNA [4].

Over the last 20 years, the development of lab-on-chip devices for performing real-time PCR (LoC-PCR) has been an active research field. LoC devices are advantageous compared to macroscopic equivalents due to their smaller dimensions, high integrability and automation, time and cost reduction, high sensitivity and selectivity, ability to integrate multistep operations in a single chip, and the possibility of real-time monitoring [5,6]. Indeed, small scale means low fluid volume for the analysis and rapid heat transfer, thus shortening the time of analysis. Compared to the standard bulky equipment, real-time LoC-PCR devices are smaller, ensuring portability and making these devices suitable for point-of-care (POC) diagnostics and in-field analysis. These features indicate that LoC-PCR may represent an important breakthrough for the early detection of pathogens and a prompter of actions to control disease epidemics and infection spreading. A LoC for DNA amplification needs to include multiple integrated components such as thermalization and temperature measurement to provide the temperature cycles, the detection element to follow the DNA amplification, and the fluidic handling device, where the amplification reaction occurs [7,8]. Reports about the development of LoC-PCR systems show different techniques for providing temperature cycles, including commercially available external heaters, heaters integrated with the fluidic handling device made of thin metallic layers and noncontact heaters based on hot-air and IR-based heating [9,10]. Noncontact heaters are enabled to carry out up to 40 PCR cycles in 370 s. For the cooling phase, these optical systems often employ air cooling through a fan or only air contact in an open system (this latter leading to PCR contamination problems); moreover, they require cumbersome setups that hinder their integration. In LoC-PCR, temperature control typically depends on the selected fluidic handling device. In general, PCR amplification can be based on static chambers or flow-through microfluidics chips [11]. In the case of static chamber, the thermalization is usually controlled by using a thermocouple [12,13] or thin-film temperature sensors made from some metallic, nonmetallic or oxide materials by using thin-film deposition techniques [9,14], and the cooling is provided by a fan. Conversely, in the flow-through systems, the amplification mix moves circularly on heaters having different temperatures, thus providing the temperature cycles [15,16]. Another method to control the thermalization in static PCR chambers is based on hot-air/liquid flows [17,18,19,20]. This technique allows very quick amplification with optimal sensitivity, but the equipment needs for liquid circulation seems bulky, compromising the portability of the LoC-PCR and thus their employment for in-field applications.

The selection of the fluidic handling for the amplification is fundamental to control the number of analyzed samples and the sample volume. Small reaction volume is advantageous because it needs less sample; on the other hand, the decreased volume may affect the limit of detection. Static fluidic chambers allow different types of designs, such as parallel multiple microfluidic channels [15,21,22] or microwell plates [23,24], enabling multiple sample analyses. Contrariwise, flow-through microfluidic chambers may analyze only one sample at a time, and it is necessary to change the channel design to obtain the different thermal profiles needed [7,11,25].

In order to implement the real-time PCR in a LoC system, it is also essential to integrate the detection system, which may be based on an optical microscope [13,26], a CCD camera [10,24,27], a smartphone [21] or on optical fibers-based systems [19]. Despite the good achievements in terms of sensitivity and speed of the amplification, which in some devices was achieved in a few minutes, in all the abovementioned real-time PCR-LoCs, the integrations of the thermalization, temperature control and detection are obtained by assembling different independent modules, making it difficult to amplify more than one sample or to obtain a portable device, in particular when an optical microscope is used. Among the industrial PCR-LoCs available on the market, many display some limits: (1) need for post-amplification amplicon characterization [28]; (2) reduced sensitivity; and/or, (3) amplification efficiency compared to commercial PCR systems; or not characterized as noted by Houssin et al. [19]. Indeed, a “true” integration of all modules to perform real-time PCR on a unique single substrate reduces the size of the equipment leading to a POC diagnostics device [23,29]. Also, some commercial LoC-PCR systems allow only the application of an isothermal amplification or are constrained to the use or dedicated cartridges, different for each type of analysis, and thus limiting the applicability [28].

Recently, we reported on the development of a system on glass (SoG) where thermalization, temperature control and fluorescent detection are all integrated on a single glass substrate [30]. This SoG was optically coupled with microfluidic chips for different applications such as isothermal amplifications of DNA [31,32] and DNA detection [33,34]. Herein, we explore the use of a new SoG based on a previously reported technology [29] to perform real-time RT-qPCR for the detection of viruses. This LoC is a portable device enabling good temperature homogeneity, a fine tracking of the temperature and the detection of the fluorescent signal resulting from the RNA amplification. In this work, the diagnostic performance of the Lab-on-Chip device was evaluated by comparison with the BIO-RAD CFX96 real-time PCR detection system. In particular, two different RNA viruses were included in this study: watermelon mosaic virus (WMV), a RNA plant virus infecting cucurbits plants, and the SARS-CoV-2 human RNA virus. For both methods, analytical sensitivity and specificity and time required on test performing have been evaluated.

## 2. Materials and Methods

### 2.1. Fabrication of the System on Glass

The system on glass (SoG) is fabricated on a single 5 × 5 cm^2^ 1-mm-thick glass (Borofloat 33 from Schott, Wolverhampton, United Kingdom). The use of a single glass substrate permits to achieve an optoelectronic platform that avoids the use of external heaters (such as Peltier cells or bulky metallic heaters) and of external long pass filters. These features lead to a very compact system, which reduces the spatial distance between the biological and electronic “worlds” increasing the system portability. A thin-film heater is deposited on the bottom side of the glass, while temperature and light amorphous silicon (a-Si:H) sensors and an interferential filter are built on the top side of the same glass. Thin-film heater, temperature and light a:Si-H sensors are fabricated using standard microelectronic technologies, as reported in the Supplementary Materials (see Sections S1 and S2, Supplementary Materials) [29,35,36]. Careful attention has been paid to the technological sequence and thin-film material selection used during the processes to maintain their technological compatibility, without degrading the performances of the previously deposited devices.

*Fabrication of the microwell plate*: The microwell plate is a 4-mm-thick black polydimethylsiloxane (PDMS) block hosting 6 round wells with diameter equal to 3 mm. The PDMS block, purchased by Finnadvance (Oulu, Finland), was bonded to a 2.8 × 3.2 cm^2^ glass slide having a thickness of 0.2 mm using the following procedure:

1. Immediately prior to use, glass slides of 2.8 × 3.2 cm^2^ were cleaned with ethanol and dried with a stream of nitrogen and subsequently irradiated for 10 min under UV/ozone (Diener Electronic, Ebhausen, Germany).
2. A PDMS mixture containing curing agent SYLGARDTM 184 silicon elastomers in a ratio of 10:1 is placed in a vacuum chamber to eliminate the air bubbles.
3. The PDMS (0.5 mL) mixture is spin-coated on a 5 × 5 cm^2^ glass slide having 1 mm thickness (previously cleaned with ethanol and dried with a stream of nitrogen) by using laurel WS-650-23 (Landsdale, PA, USA).
4. The bottom side of the black PDMS microwell is placed on the PDMS spin-coated glass slide for a few minutes. Subsequently, it is removed and placed on the ultraclean 0.2 mm glass slides.
5. The glass-PDMS microwell is placed in the vacuum chamber for at least 10 min to remove the gas bubbles which may form between glass and PDMS during the bonding. After degassing, the glass-PDMS microwell is heated at 100 °C for 30 min to finalize the bonding.

**Figure 1:**
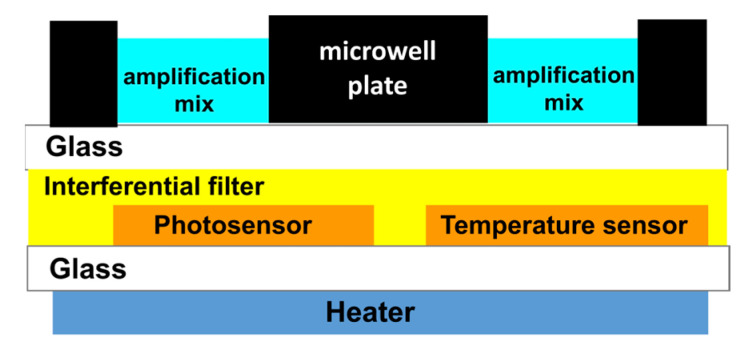
Scheme of the coupled SoG and microwell plate.

The SoG and microwell plate are optically and thermally coupled to obtain the LoC system for performing the real-time RT-qPCR, as schematically shown in Figure 1.

The PDMS has been chosen as the material for the microwell plate thanks to its biocompatibility and easy and rapid fabrication process. Furthermore, the bonding to a very thin glass substrate allows high transparency for the fluorescent radiation, and at the same time, reduces the distance between the photosensors and the reaction well, where the fluorescence is generated. Moreover, a-Si:H sensors can be deposited on glass substrate according to their low deposition temperature (around 200 °C), ensuring high performances both in terms of sensitivity and stability [33,35].

**Figure 2:**
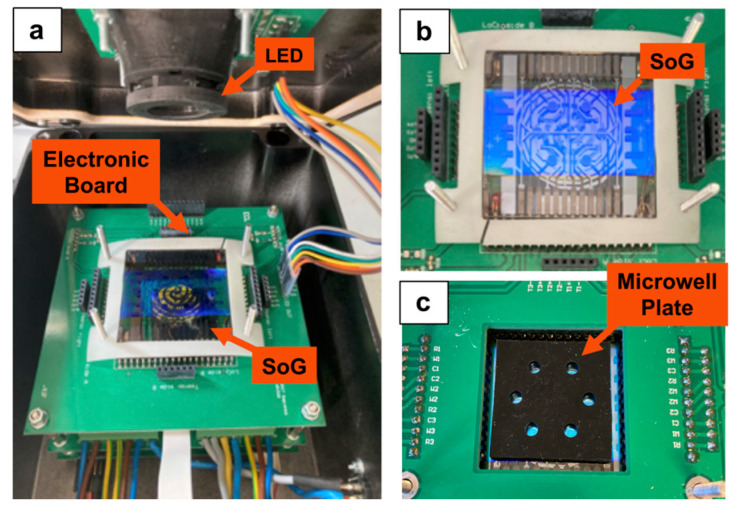
Image of the LoC (**a**) metallic box containing the light-emitting diode (LED) anchored to the box lid, the electronic board and the SoG, (**b**) top view of the SoG and (**c**) microwell plate optically aligned with the SoG.

The LoC is connected to electronic boards and enclosed in a portable metallic box having the following dimensions: 30 × 20 × 12 cm^3^ and less than 2 kg of weight. The lid of the metallic box hosts a blue-light-emitting diode (APG2C1-450 from Roithner Lasertechnik GmbH, Wiedner Hauptstraße 76, A-1040 Vienna) for fluorescence excitation, which is filtered with a band-pass filter to shrink the excitation spectrum (Figure 2). The LoC is connected to a computer via a USB cable, and the photocurrent signals are visualized by a homemade software.

### 2.2. WMV Diagnosis

#### 2.2.1. RNA Extraction

The samples’ set belongs to CREA-DC collection (positive and negative certified reference material) and includes target (WMV-infected plants) and three nontarget samples (isolates of two different viral species that infect cucurbits plants: zucchini yellow mosaic virus (ZYMV) and cucumber mosaic virus (CMV), including one sample from healthy zucchini plant). The total RNA of plant samples were extracted from leaf tissue macerated in 1:5 (*w*/*v*) of 0.1 M phosphate buffer using the commercial RNeasy Plant Mini Kit (Qiagen, Milan, Italy). Briefly, 100 μL of the macerated leaf tissue were added to 380 μL of the RLT buffer provided in the kit. Then, the manufacturer’s instructions were followed (https://www.qiagen.com/us/products/discovery-and-translational-research/dna-rna-purification/rna-purification/total-rna/rneasy-plant-mini-kit, accessed on 3 May 2023). The RNA extracts were aliquoted and two sets of aliquots were prepared and stored at −20 °C to ensure the same sample conditions (concentration and handling) in both test diagnostic systems. Analytical sensitivity was evaluated using a 10-fold dilution series prepared by diluting the macerated leaf tissues of infected WMV-36 isolate in macerated healthy leaf tissues of zucchini plant by using in both the BIO-RAD XF96 and the LoC-PCR systems. The specificity was valuated using nontarget viruses: the cucumber mosaic virus (CMV) and the zucchini yellow mosaic virus (ZYMV).

#### 2.2.2. Real-Time RT-PCR by BIO-RAD CFX96

The tests were first performed on a CFX96 thermocycler (Bio-Rad) using a TaqMan™ RNA-to-CT™ 1-Step Kit (Thermo Fisher Scientific, Milan, Italy). The amplification was performed by using 1 µL of RNA extract in a mixture containing 5 µL of 2× MasterMix, 0.25 µL of 40× RT Enzyme, 0.3 µM of each primer: WMV-CP-93-F 5′-TGGGCAGGGTAGCAAGGA-3′ and WMV-CP-161-R 5′-CCTTTTGATCCAACGTTCACATC-3′ and 0.2 µM of labeled TaqMan^®^ probe, 6-Fam-CCAACAAAAGCTGGCACAGTCAGCAA-BHQ-1 [37], and PCR-grade nuclease-free water for a total reaction volume of 10 µL. In all the experiments, the amplification included an RT step at 45 °C for 30 min, an initial denaturation step at 95 °C for 10 min, and 40 cycles of denaturation and annealing/elongation at 95 °C for 15 s and 60 °C for 1 min, respectively.

#### 2.2.3. Real-Time RT-PCR-LoC System

The amplification was performed by using 1 µL of RNA extract, 2.5 µL of the 2× Sybr master mix from applied biosystems (Thermo Fisher Scientific), 0.15 µL of both 10 µM forward and reversed primers (mentioned above), 0.1 µL of AMV reverse transcriptase (Promega, Fitchburg, WI, USA), 0.15 µL of 0.1 mM [Ru(phen)_2_(dppz)]^2+^ fluorescent dye and 1.95 µL of PCR-grade nuclease-free water in a total reaction volume of 5 µL. In all the experiments, the amplification conditions were 45 °C for 15 min, 95 °C for 3 min and 35 cycles of 95 °C for 20 s and 60 °C for 1 min, followed by a melt curve from 60 °C to 95 °C in the increment of 0.5 °C/s. A no-template control (NTC) of PCR-grade nuclease-free water was included in all tests to monitor possible contaminations.

### 2.3. SARS-CoV-2 Diagnosis

#### 2.3.1. RNA Extraction

In vitro SARS-CoV-2 (Human 2019-nCoV strain 2019-nCoV/Italy-INMI1, Rome, Italy used as positive biological material control) infections (0.01 MOI) were performed on VeroE6 cells, and RNA was extracted from six days post-infection (dpi) supernatants using the Maxwell^®^ RSC Instrument with Maxwell^®^ RSC Viral Total Nucleic Acid Purification (Promega, Fitchburg, WI, USA). The RNA extracts were aliquoted, and two sets of aliquots were prepared and stored at −20 °C to ensure the same sample conditions (concentration and handling) in both test diagnostic systems. A 10-fold dilution series prepared by diluting the extracted SARS-CoV-2 RNA was used to evaluate the analytical sensitivity in both the BIO-RAD XF96 and the LoC-PCR systems. A RNA extracted from the respiratory syncytial virus (RSV) was included as a nontarget sample.

#### 2.3.2. Real-Time RT-PCR by the BIO-RAD CFX96 and LoC System

The amplification of Nucleocapsid (N) SARS-CoV-2 sequence was performed by using 4 µL of RNA, 5 µL of 2× Sybr master mix from Promega, 1 µL of 10 µM solution of forward and reverse primers (N1-F: 5′-GAC CCCAAAATCAGCGAAAT-3′; N1-R: 5′-TCTGGTTACTGCCAGTTG AATCTG-3′), 0.2 µL of reverse transcriptase (Promega), and 1 µL of PCR-grade nuclease-free water. The amplification in the LoC system was performed using 1 µL of RNA, 3 µL of 2× Sybr master mix from Promega, 0.5 µL of 10 µM solution of forward and reverse primers mentioned above, 0.1 µL of reverse transcriptase (Promega), 0.15 µL of 0.1 mM [Ru(phen)_2_(dppz)]^2+^ fluorescent dye and 0.35 µL of PCR-grade nuclease-free water. A no-template control (NTC) of PCR-grade nuclease-free water was included in all the tests to monitor possible contaminations. In all the experiments, the amplification conditions were 45 °C for 15 min, 95 °C for 3 min and 40 cycles (35 cycles for the on-chip experiments) of denaturation at 95 °C for 20 s and annealing/elongation at 60 °C for 1 min, followed by a melt curve from 60 °C to 95 °C in the increment of 0.5 °C/s.

Efficiency (E) was calculated using the following formula: E = (−1 + 10^(1/slope)^) × 100. For each virus, two of the amplified samples from both the LoC system and the standard CFX96 thermocycler were analyzed by performing gel electrophoresis in the following manner: to 5 µL of amplified sample were added 2 µL of 6× loading dye (Invitrogen) and 4 µL of MilliQ water. The samples were run on a 1.5% agarose gel containing ethidium bromide.

## 3. Results and Discussion

The PCR-LoC presented here (Figure 2) is a portable system that can be used for DNA/RNA amplification and detection. The LoC features a high miniaturization degree thanks to the on-chip detection performed by the a-Si:H photosensors and the short distance between the fluorescent signal and a-Si:H photosensors sites avoids the need for focusing optics. The PCR-LoC system is tested for the detection of two different RNA viruses, WMV-infecting cucurbits plants and SARS-CoV-2. Both viruses are also tested using the BIO-RAD CFX96 real-time PCR detection system. The efficiency of the PCR-LoC device in virus diagnosis is evaluated by comparison of data obtained on analytical sensitivity, analytical specificity and times required in test performing from both diagnostic systems.

The detection of RNA viruses is performed by using the one-step real-time RT-qPCR. In this method, in the same amplification mixture, the RNA is first transcripted to c-DNA, which is then amplified through the PCR reaction. The SoG provides the thermal cycles required by the PCR through the combined action of heater and temperature sensors and the detection of the fluorescent signal deriving by DNA amplification through the a-Si:H photosensors. The PCR reaction occurs in the microwell plate optically and thermally combined with the SoG. The microwell plate can host six samples, each having a volume of up to 30 µL. The LoC has been designed for detecting the natural fluorescence of the ruthenium complex [Ru(phen)_2_(dppz)]^2+^, a DNA intercalating dye. Its luminescence is poor in aqueous solutions but shows a sharp increase when the molecule is intercalated into the dsDNA. This fluorophore has a Stokes shift of about 150 nm [38]. This optical property allows to fabricate a more efficient interferential filter, providing the almost complete cutoff of the light deriving from the excitation. The thin-film interferential filter deposited on the top of the SoG has to transmit only the emission spectrum of the [Ru(phen)_2_(dppz)]^2+^ complex, rejecting at the same time the excitation wavelengths. This reduces the photosensor background signal and avoids the saturation of the readout electronics. As the DNA amplification proceeds, the resulting increase of the fluorescent signal is detected by the a-Si:H photosensors positioned underneath. The increase of the fluorescent intensity is detected as the average photocurrent measured during the annealing/elongation step of the amplification at 60 °C for both viruses (see Figure S5).

### 3.1. Temperature Generation and Control on the SoG

**Figure 3:**
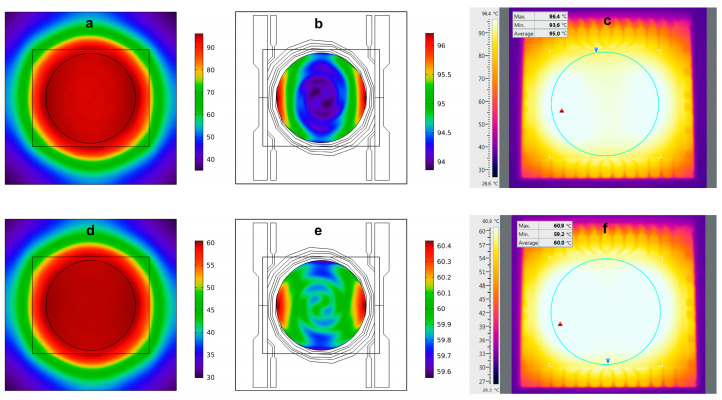
Modeled temperature distribution (**a**) at 95 °C over the whole 5 × 5 cm^2^ glass substrate, (**b**) at 95 °C over the active area of the heater, (**d**) at 60 °C over the whole 5 × 5 cm^2^ glass substrate, (**e**) at 65 °C over the active area of the heater (circle with a 2.8 cm diameter). Temperature distribution measured for set temperatures of 95 °C (**c**) and 60 °C (**f**). The blue circles enclose the active area of the heater, while the red and blue triangles represent the highest and lowest temperatures detected, respectively.

The LoC allows to set different thermic profiles as necessary, according to the denaturation and annealing/elongation temperatures of the selected primers and the master mix, respectively. Initially, the heater performance has been tested using modeled 2D thermal maps achieved with COMSOL Multiphysics at 95 °C and 60 °C, as reported in Figure 3. We have chosen these specific temperatures as they are those selected in the temperature profile of the RT-qPCR for RNA amplification of the WMV and SARS-CoV-2. We observed that, even though the whole temperature distribution (Figure 3a) ranges between 35 °C (in the glass corners) and 96.5 °C (in the warmest part of the SoG), the temperature variation is only 2 °C in the active region of the region (the area inside the circle), as shown in Figure 3b. As expected, temperature distribution at 60 °C is better than that at 95 °C. It ranges between 30 °C and 60 °C in the whole glass substrate, while it varies by only 1 °C in the active area of the heater (Figure 3d,e).

In the active area of the heater, we observe that, at 60 °C and 95 °C, the maximum displacement is 0.9 °C and 1.4 °C, respectively. These data showed a maximum temperature variation of 1.7 °C and 2.8 °C at 60 °C and 95 °C, respectively. Even though these variations are slightly worse than the theoretical data shown in Figure 3, they do not compromise the successful implementation of the real-time PCR technique as is demonstrated below. The larger variations of the measured temperatures with respect to the modeled ones can be ascribed to the local variations in thickness and width of the turns of the thin-film heater.

**Figure 4:**
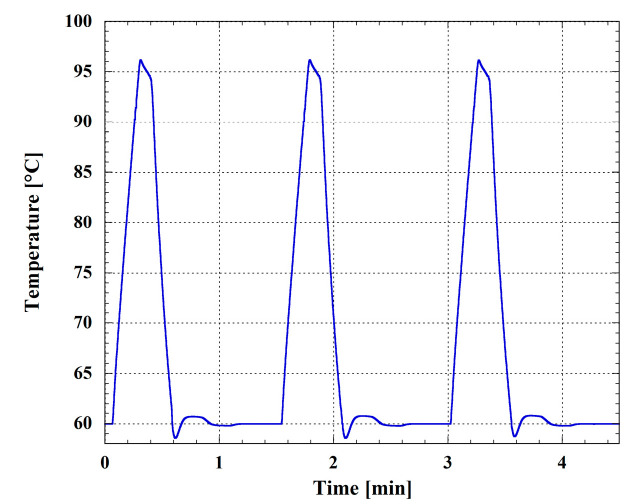
Temperature profile of three temperature cycles in the SoG coupled with the glass-PDMS microwell plate as measured by the a-Si:H temperature sensor.

In order to test the effectiveness of the temperature control of the system, we performed a preliminary temperature cycling, varying the temperature between 60 °C and 95 °C in the operating conditions of the system, i.e., with the microwell plate placed over the SoG. Figure 4 reports the temperature measured by one of the temperature sensors integrated in the SoG. The heating rate is 2.2 °C/s, and each cycle, for the selected temperature profile for the RNA amplification, takes 1.5 min. The cooling rate is about 2.2 °C/s and is provided by a fan integrated in the metallic box under the SoG.

### 3.2. Optimization of Reaction Conditions

**Figure 5:**
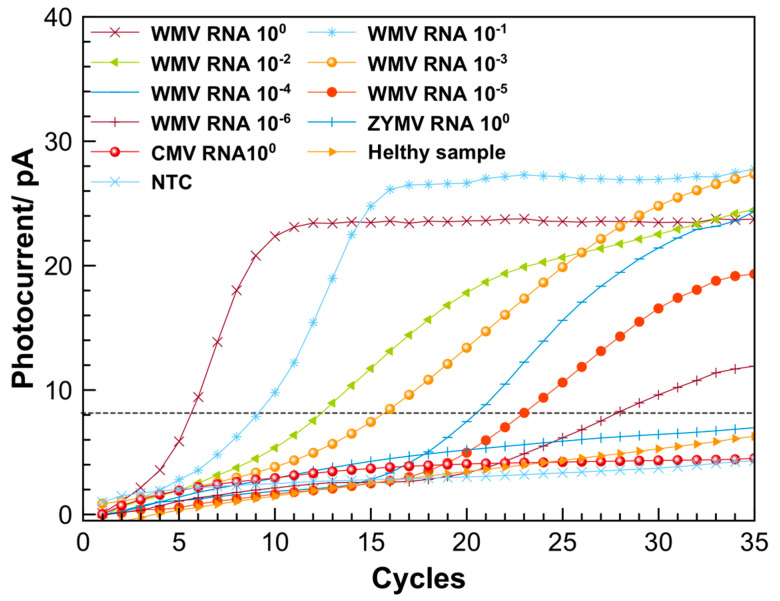
Amplification curves from testing the 10-fold dilution series of extracted RNA from WMV-infected sample, performed by using the LoC system (amplification curves are an average of three repeated measurements).

The glass-PDMS microwell plate (Figure 2c) is of a block of black PDMS having six wells with a volume of up to 30 µL bonded to a glass slide. The wells were designed to be optically aligned with the array of a-Si:H photosensors. The black color of the PDMS block was necessary to prevent light cross-contamination between the neighboring photosensors, as was observed when transparent PDMS was used. In order to perform the real-time RT-PCR, the microwell plate is positioned above the SoG, and the wells are optically aligned with the photosensors. Subsequently, 4 µL of PCR mixture are dispensed in each well, and 1 µL of the extracted RNA is added. The spotted volume permits that, once the sample is spotted in the microwell, the liquid would form a droplet on the glass surface without touching the PDMS wall of the microwell plate. Afterwards, 11 µL of mineral oil are added to cover the solution acting as a surrounding droplet, as the oil wetting the hydrophobic PDMS inner surface of the well. This procedure avoids both the evaporation of the solution and air bubble formation due to the porosity of PDMS. The microwell plate is kept in contact with the SOG by means of a homemade holder, which ensures uniformity of the heats on the glass surface of the microwell plate, as was observed by the thermocamera (Figure 5).

Amplification and detection of WMV and SARS-CoV-2 sequences. Initially, the LoC was used for the detection of WMV. Figure 5 presents the amplification curves obtained by performing the real-time RT-PCR using the LoC system. The curves were obtained by elaboration of the original amplification signals using a homemade software; an example of the row data and description of elaboration procedure are reported in Figure S5. During the amplification experiments, an increase of the photocurrent, starting at different cycle numbers was observed down to 10^−6^ RNA sample dilution. In order to verify any possible cross-contamination, one of the wells was used as a blank control (NTC), where only water was added to the RT-PCR mixture. The amplification curve resulting from the NTC sample showed an increase of photocurrent much lower with respect to the samples containing RNA, for all the dilutions considered for the amplification. This confirmed the reliability of the LoC for performing the real-time RT-qPCR.

The specificity of the RT-qPCR was proved by performing the amplification using RNA extracted from the cucumber mosaic virus (CMV) and the zucchini yellow mosaic virus (ZYMV), and using the extraction matrix solution from a healthy zucchini plant (healthy sample). As can be observed in Figure 5, these three curves did not show any amplification signal.

Figure 5 displays that, after 30 cycles, all RNA samples exhibit an increase of the photocurrent signals down to the 10^−6^ dilution factor. The RT-qPCR of the RNA samples was also performed using the CFX96 thermocycler (see Figure S4). The melt curve analysis performed by using the LoC resulted in a melting temperature of (79 ± 2) °C (an example of the melting curves is reported in Figure S6), according with the melting temperature of (80 ± 0.5) °C obtained by the thermocycler.

**Figure 6:**
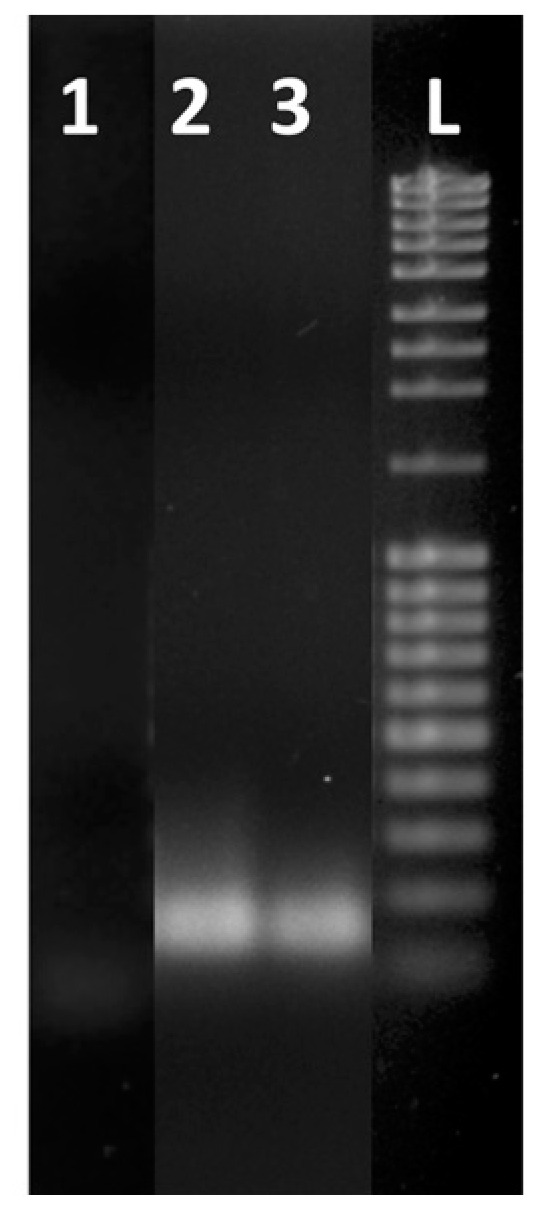
Gel electrophoresis of real-time RT-PCR products after 35 cycles of amplification: blank (lane 1), amplification products from extracted RNA of WMV-36 isolate sample using a CFX96 PCR detection system (lane 2) and using LoC (lane 3). Lane L contains a 100 bp DNA ladder.

The samples amplified by using the LoC and the thermocycler (concentration = 10^0^) were analyzed by gel electrophoresis (Figure 6) to confirm that the increase of the photocurrent observed in Figure 5 was due exclusively to the formation of the specific amplicons. The gel electrophoresis showed the presence of the same amplicons for both the on-chip- and CFX96-amplified samples (Figure 6).

The RT-qPCR efficiency for the on-chip amplification was also evaluated. For this purpose, a threshold was set at 8 pA (dashed line in Figure 5), considering the statistically significant increase over the baseline signal obtained with the NTC sample. The calibration curve (see Figure S3) was obtained plotting the cycle values (Cq), corresponding to the intersection between the threshold line and the amplification curves, as a function of the RNA dilution factors. The data were fitted with a straight-line equation (y = 3.44x + 6.23, R^2^ = 0.999, where x represents the logarithmic value of RNA dilution and y the resulting Cq), and the slope was used to calculate the PCR efficiency, which resulted to be 95.5%. This value is comparable with the efficiency of 99.66% obtained by performing the amplification using a previously validated protocol for the WMV diagnosis by the CFX96 thermocycler [37] (see Figure S4).

**Figure 7:**
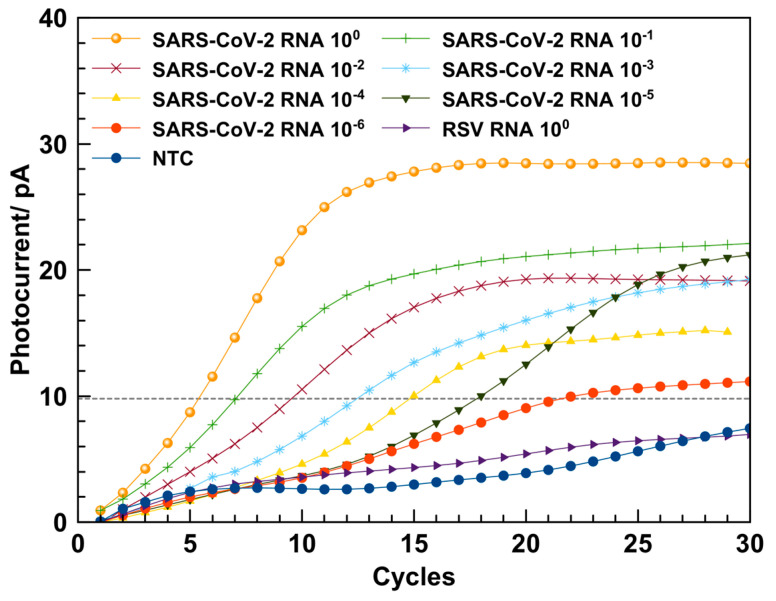
Amplification curves obtained from the amplification of 10-fold dilution series of RNA from SARS-CoV-2 performed by using the LoC system (amplification curves are an average of three repeated measurements).

In order to show the applicability of the above described LoC-PCR for detection of different viruses, it was also used for the amplification of the RNA extracted from SARS-CoV-2 samples. In Figure 7 are reported the amplification curves obtained for the 10-fold dilution samples. A NTC sample was used to verify possible cross-contamination occurring during the amplification. The variation of photocurrent observed for the NTC sample was lower compared to the samples containing RNA, for all the dilutions considered for the amplification, thus confirming the consistency of the LoC used for the LoC RT-qPCR. The specificity of the RT-qPCR was proved by performing the amplification using RNA extracted from the respiratory syncytial virus (RSV), which did not show an amplification signal (Figure 7). The melt curve analysis performed using the LoC resulted in a melting temperature of (80 ± 2) °C (an example of the melting curves for SARS-CoV-2 is reported in Figure S6), in accordance with the melting temperature of (82.5 ± 0.5) °C obtained by the thermocycler.

**Figure 8:**
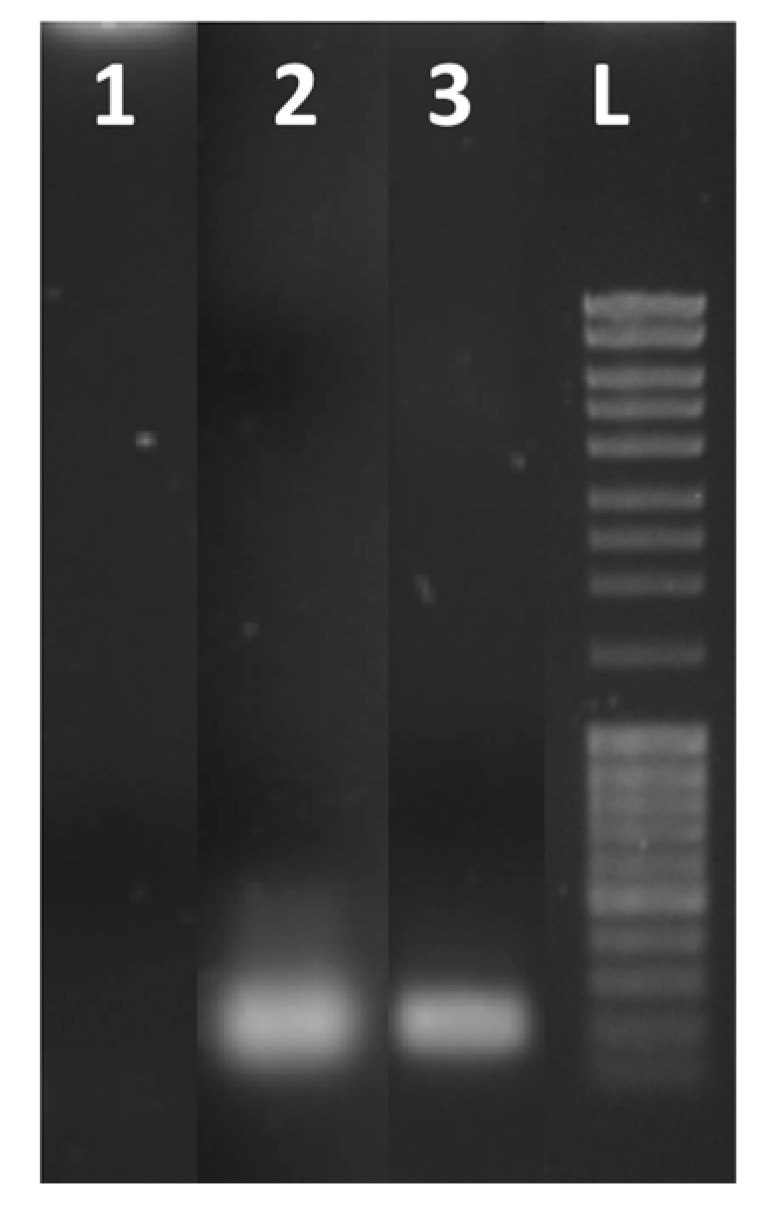
Gel electrophoresis of real-time RT-qPCR products after 35 cycles of amplification: blank (lane 1), RNA from SARS-CoV-2 using a CFX96 real-time PCR detection system (lane 2) and using the LoC-PCR (lane 3). Lane L contains a 100 bp DNA ladder.

The RT-qPCR of the RNA samples was performed also using the CFX96 thermocycler (see Figure S4-2). The analysis of the amplification products by gel electrophoresis showed that the RNA amplification on-chip leads to the formation of one amplicon, as was also observed in the amplification by using the CFX96 thermocycler (Figure 8).

Also for this experiment, a calibration curve (see Figure S3) was achieved plotting the Cq as a function of the RNA dilutions factors, considering the threshold at 10 pA (Figure 7). The data were fitted with a straight-line equation (y = 2.94x + 5.548, R^2^ = 0.999), and the slope was used to calculate the PCR efficiency, which resulted to be 115.4%. This value is comparable with the efficiency of 92.3% obtained using the CFX96 real-time amplification system within the experimental error (see Figure S4).

The results obtained for the RNA amplification of both viruses suggest that the efficiency of the real-time RT-PCR performed by using the LoC-PCR and CFX96 thermocycler are all in the acceptable amplification efficiency range of 90–120% [39]. It is also worth comparing the total time necessary to conduct the amplification. As a matter of fact, to detect the 10^−6^ diluted sample, 60 min and 120 min are needed for the on-chip and off-chip measurement, respectively (including the reverse transcription step and the melting curve analyses). This is a consequence of two intrinsic characteristics of the Lab-on-Chip. The first is connected to the small dimensions of the LoC-PCR: as a matter of fact, the duration of a single cycle is 1.5 and 2.5 min with a heating speed of 2.2 and 4 °C/s for the LoC-PCR and the CFX96 thermocycler, respectively. This means that, although the heating speed is slower for the LoC-PCR, the duration of a single cycle is shorter. This result can be ascribed to the faster heat transfer from the heater to the temperature sensor and the microwell in the LoC-PCR, due to its smaller dimensions. Secondly, the fluorescence signal derived from the amplification is detected earlier in the LoC-PCR. As shown in Figure 5 and Figure 7, for the same sample dilutions, the threshold cycles values (Ct) obtained for the on-chip amplification are on average 5 cycles in advance compared to those displayed by using the CFX96. This effect can be explained by the short distance between the photosensors and the microwell plate, confirming that the integration of thermalization elements and the photosensors on the same glass substrate improves the detection of the fluorescence signal and thus the overall LoC-PCR time of performance, which is comparable with other integrated devices applied for real-time PCR [13,23]. In terms of sensitivity, this device showed similar results to other “true integrated” LoC-PCR, which were demonstrated as having the same limit of detection achievable using standard thermocyclers [40,41].

## 4. Conclusions

In this work, a LoC-PCR system consisting of a SoG, where thermalization, temperature control and photosensors for fluorescence detection all integrated on a single substratum named SoG, is coupled with a microwell plate, where the molecular reaction occurs. The LoC-PCR was tested for the detection of the WMV and SARS-CoV-2, a plant and human virus, respectively, by using real-time RT-PCR. We demonstrated that the LoC-PCR can be used for quantitative analysis, and the limit of detection and the efficiency were calculated. These values were comparable with those obtained using the CFX96 standard thermocycler. However, the performance of the LoC-PCR shows that the amplification time is comparable to that of other “true integrated” PCR-LoC [23] and improved compared to the CFX96, as half-time is needed for RNA detection. The LoC-PCR requires a 5 µL reaction instead of the 10 µL used by the standard machine, and the RNA is detected, on average, 5 Ct earlier than by using CFX96. The dimension of the system allows portability and provides the possibility to test up to six samples, which is higher compared to other portable devices [21,23]. In further work, we aim to conduct validation experiments where specificity and accuracy can be evaluated, analyzing several samples of non-specific RNA/DNA, as well as RNA/DNA extracts from healthy and sick plants or patients, respectively. Due to its flexibility, we envy to combine this LoC-PCR with other components for sample treatment [20,42], i.e., a device for DNA/RNA extraction, leading to a stand-alone device for a wide type of biomolecular analysis.

## Supplementary Materials

The following supporting information can be downloaded at: https://www.mdpi.com/article/10.3390/bios13050544/s1, Section S1: Fabrication of the thin-film heater of the LoC; Section: S2: Fabrication of Amorphous silicon photosensors (a-Si:H); Section S3: Real time RT-qPCR efficiency obtained by using the LoC-PCR system; Section S4: Amplification curves and real time RT-qPCR efficiency obtained by using the CFX96 thermocycler; Section S5: Elaboration of the data obtained from the on-chip real time RT-qPCR; Section S6: Elaboration of the data obtained from the on-chip real time RT-qPCR.
