# Supplementary material for: Biomolecular Monitoring Tool Based on Lab-on-Chip for Virus Detection
Source: Biosensors (Basel). 2023 May 12;13(5):544. doi: 10.3390/bios13050544 (PMC10216243; doi:10.3390/bios13050544)
Supplement: Supplementary file 1 [file biosensors-13-00544-s001.zip › biosensors-2315069-supplementary.pdf]

# Biomolecular Monitoring Tool Based on Lab-on-Chip for Virus Detection

## Supplementary Materials

### S1 Fabrication of the thin film heater of the LoC

The heater is a chromium (Cr)/aluminum (Al)/chromium (Cr) metal stack deposited by a vacuum evaporation system (Balzers Mod. 510). The turn thicknesses and spaces of its double spiral geometry (Figure S1) have been optimized with Comsol Multiphysics in order to achieve a uniform temperature distribution over an active area of about 6 $cm^2$.

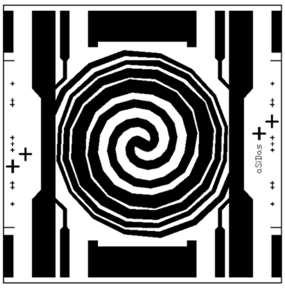

**Figure S1:** Optimized geometry of the thin film heater. The double spiral provides the active region of the heater, the wide vertical lines are the electrical contact, while the thin vertical lines can be used in a four-tips measurement for reading the voltage across the resistor. Crosses on the glass provide the reference marks for all the photolithographic steps.

The detailed fabrication process of the heater is reported in detail in a previously reported publication (Lovecchio et al., 2022) and it is briefly summarized as following:

a. cleaning of the glass substrate with piranha solution;
b. deposition, by vacuum evaporation, of the Cr/Al/Cr (with thickness equal to 30, 600, 70 nm, respectively) stacked layer;
c. patterning of the stack metal layer to define the thin film heater geometry (reported in Figure 2) by means of standard optical lithography and wet etching processes;
d. deposition of a passivation layer through the spin coating of a 5 µm-thick SU-8 3005 (from MicroResist, Berlin, Germany).

Optical lithography has been accomplished by using a mask aligner (ORIEL Coroporation, Stratford, CT), the photoresist AZ 15-18 and its developer and remover (MicroChemicals GmbH, ULM, Germany).

In order to set a precise temperature over the active area of the glass substrate, an electronic board supplies the current to the heater according to a Proportional-Integral-Derivative (PID) algorithm which receives as inputs both the set-temperature and the output of the temperature sensors (Lovecchio et al., 2022).

### S2 Fabrication of Amorphous silicon photosensors (a-Si:H)

The temperature and photo-sensors are p-type/intrinsic/n-type hydrogenated amorphous silicon (a-Si:H) structures grown by Plasma Enhanced Chemical Vapor deposition and patterned through optical photolithography. They are deposited on the opposite side of the glass substrate with respect to the thin film resistor. There are eight photosensors on a radial geometry and six

temperature sensors close to the photosensors as shown in Figure S2. The same figure shows the heater, in order to show the alignment of the optoelectronic devices on the SoG.

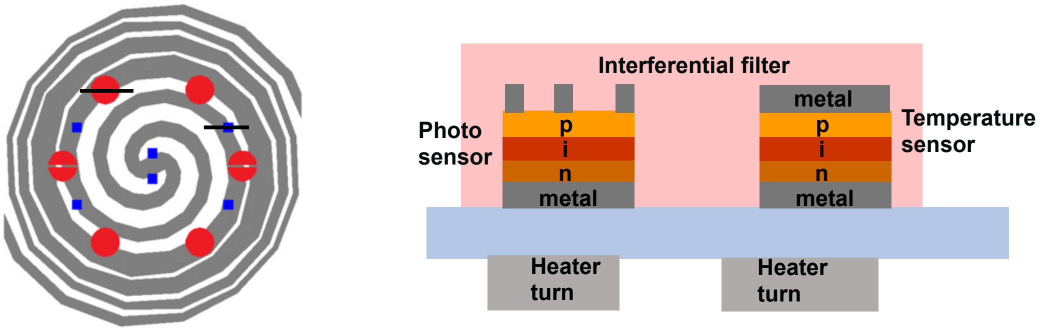


**Figure S2.** Schematic top view (left) and cross section (right) along the two black lines of the devices fabricated on the SoG. The interferential filter fabricated on top of the a-Si:H sensors rejects the excitation light and transmits only the fluorescent signal.

The whole fabrication process of the is reported in detail in [reference]. Briefly, here we report the fabrication steps of the a-Si:H sensors:

1. deposition by vacuum evaporation of a Cr/Al/Cr (30/150/30 nm) stacked layer and its patterning to define the sensors bottom contacts;
2. deposition of the a-Si:H layers by means of a Plasma Enhanced Chemical Vapor Deposition system (Glasstech Solar Incorporation, Denver, Colorado);
3. deposition by vacuum evaporation of a 50 nm-thick Cr layer, working as the top contact;
4. patterning of the metal layer and the a-Si:H layers by wet etching and reactive ion etching, respectively to define the sensors. Dimensions of the temperature sensors are 1.0 x 1.0 $mm^2$, while the photosensors have a diameter equal 3mm;
5. deposition by spin coating of a 5 µm-thick SU-8 3005 passivation layer and its pattering for opening via holes over the diodes;
6. deposition by magnetron sputtering of a 150 nm-thick titanium/tungsten alloy layer and its patterning for the definition of the top contacts and of the connection to the pad contacts;
7. deposition by spin coating of a 5 µm-thick SU-8 3005 passivation layer.

The interferential filter is a long-pass filter constituted by alternating layers of $TiO_2$/$SiO_2$ dielectric layers, whose thicknesses have been optimized to reject wavelengths below 510 nm and transit without attenuation (below 5%) wavelengths above 600 nm (Lovecchio et al. IEEE Trans. Biomed. Circuits Syst. 2018, 12, 1337–1344,). It has been designed with a freeware software (XOP with the IMD extension) and deposited by using the electron beam physical vapor deposition technique without any patterning process. The filter deposition was performed at room temperature, to preserve the functionality of the photosensors previously fabricated on the same substrate.

In order to read-out the sensor photocurrents, a low-noise electronic board based on charge sensitive amplifier has been employed [*Nascetti, A., Colonia, G., Caputo, D., & De Cesare, G. S. A general purpose sub-picoamps current readout electronics. In Lecture Notes in Electrical Engineering, Proceedings of the 2nd National Conference on Sensors, Rome, Italy (pp. 19-21)]*.

## S3 Real time RT-qPCR efficiency obtained by using the LoC-PCR system

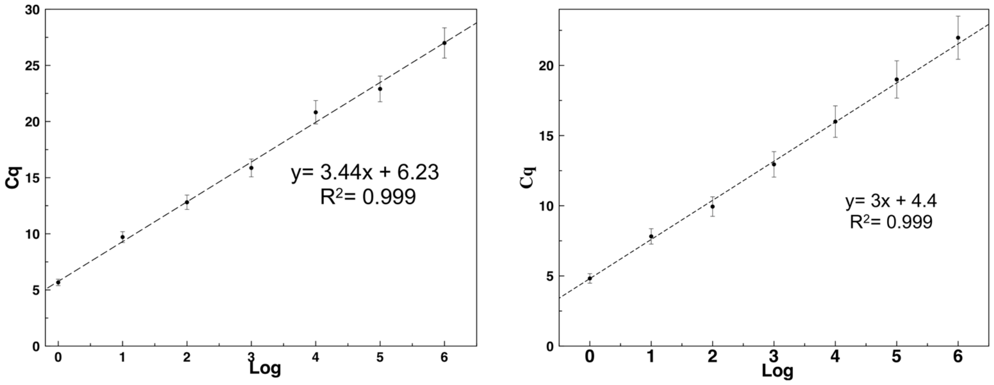


**Figure S3.** Calibration curve for the on-chip RT-qPCR of WMV (left) and SARS-CoV-2 (right) RNA. The quantification cycle (Cq) value is plotted against the log of RNA ten-fold serial dilution. The value of the slopes reported in the straight-line equation allowed for the estimation of the efficiency of the reaction (y= 3.44x + 6.23, $R^2$= 0.999 for WMV and y= 2.94x + 5.548, $R^2$= 0.999 for SARS-Cov-2). Error corresponding to the relative standard deviation was 8% and was calculated over three on-chip amplification experiments (n=3).

## S4 Amplification curves and real time RT-qPCR efficiency obtained by using the CFX96 thermocycler

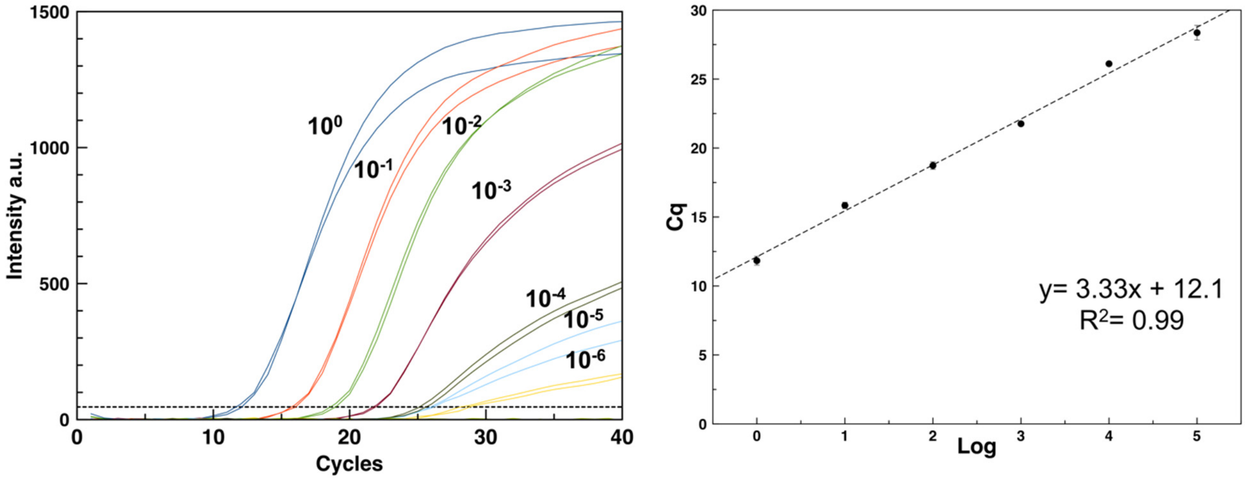


**Figure S4:** (left) amplification curves from testing the 10-fold dilution series of RNA from WMV performed by using the CFX96 thermocycler and (right), efficiency was calculated to be 99.66%.

The melting temperature of the WMV primers was 80 $\pm$ 0.5 °C.

The melting was tested performing the real time RT-qPCR by using the CFX96 thermocycler: 1 µL of RNA from WMV, 5 µL of the 2X Sybr master mix from applied biosystems (Thermo Fisher Scientific) 0.3 µL of both 10 µM forward and reversed primers, 0.25 µL AMV reverse transcriptase, and 4 µL of PCR-grade nuclease free water in a total reaction volume of 12 µL. Temperature profile: 40 cycles of denaturation and annealing/elongation at 95 °C for 15 s and 60 °C for 1 min respectively. Melt curve from 60 °C to 95 °C in the increment of 0.5 °C/ s.

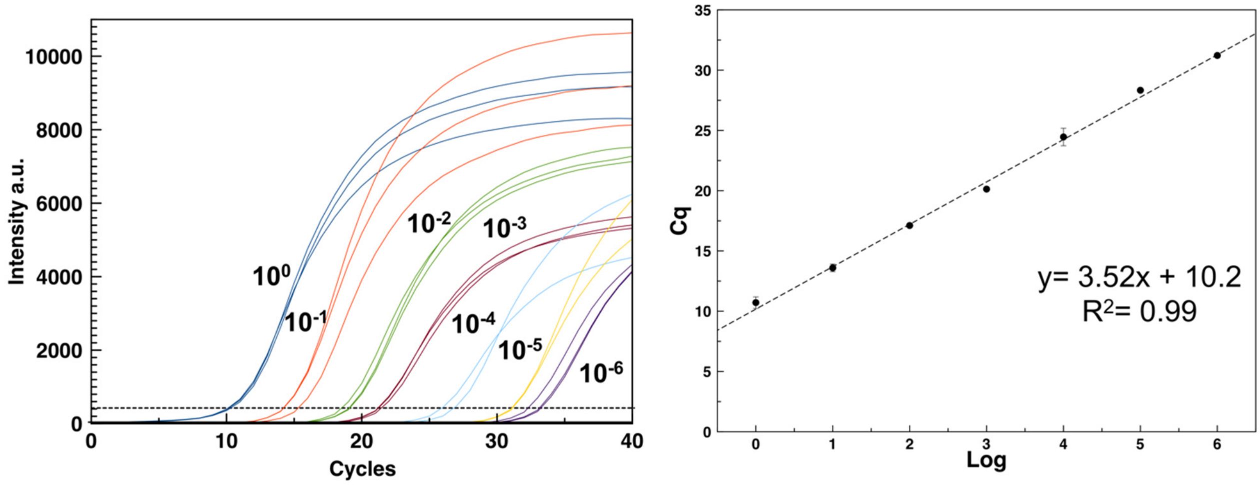


**Figure S4-2:** (left) amplification curves from testing the 10-fold dilution series of RNA from SARS-CoV-2 performed by using the CFX96 thermocycler and (right), efficiency was calculated to be 92.35% (melting temperature was 82.5 ± 0.5 °C).

## S5 Elaboration of the data obtained from the on-chip real time RT-qPCR

Each point of the amplification curves showed in figure 5 and 7 was obtained using an home-made software. Hundred data points which were obtained from the fluorescent signal during the annealing/amplification step at 60 °C (see Figure S5), for each cycle, were averaged to achieve a single data point. Each data point was then normalized using the following formula $(P_1\text{-}P_0)/\ P_0$, where $P_0$ is the photocurrent of the first cycle at 60 °C and $P_1$ is the photocurrent for all the other cycles at 60 °C. The data obtained for each cycle was used to build up the amplification curves reported in figure 5 and 7.

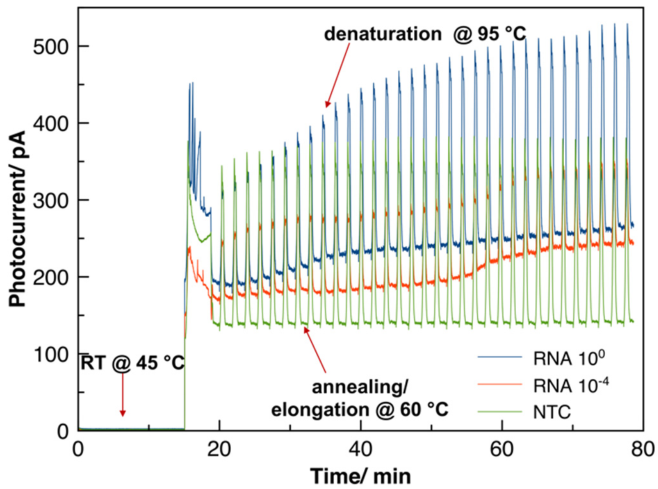


Figure S5: Example of amplification curves obtained during the real time RT-PCR performed on-chip for the RNA of WMV.

## S6 Melting Curves of the real-time RT-PCR performed using te LoC-PCR system

The melting curves were performed using the following temperature: 60 °C to 95 °C in the increment of 0.5 °C/ s.

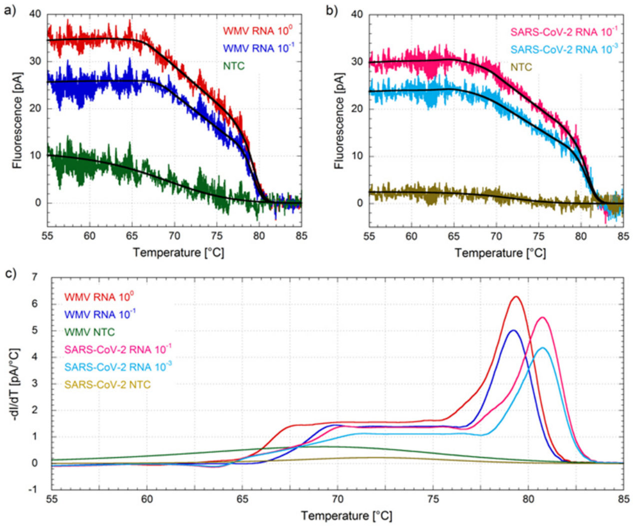


**Figure S6:** Example of melting curves obtained using the LoC-PCR system. Decrease of the fluorescence in function of temperature for **a)** WMV and **b)** SARS-CoV-2, and **c)** derivatives obtained from the curved fitted in **a)** and **b)**.
